# Low Levels of Genetic Divergence across Geographically and Linguistically Diverse Populations from India

**DOI:** 10.1371/journal.pgen.0020215

**Published:** 2006-12-22

**Authors:** Noah A Rosenberg, Saurabh Mahajan, Catalina Gonzalez-Quevedo, Michael G. B Blum, Laura Nino-Rosales, Vasiliki Ninis, Parimal Das, Madhuri Hegde, Laura Molinari, Gladys Zapata, James L Weber, John W Belmont, Pragna I Patel

**Affiliations:** 1 Department of Human Genetics, Bioinformatics Program, and the Life Sciences Institute, University of Michigan, Ann Arbor, Michigan, United States of America; 2 Institute for Genetic Medicine, Keck School of Medicine, University of Southern California, Los Angeles, California, United States of America; 3 Department of Neurology, Baylor College of Medicine, Houston, Texas, United States of America; 4 Department of Molecular and Human Genetics, Baylor College of Medicine, Houston, Texas, United States of America; 5 Center for Medical Genetics, Marshfield Medical Research Foundation, Marshfield, Wisconsin, United States of America; 6 Center for Craniofacial Molecular Biology, School of Dentistry, University of Southern California, Los Angeles, California, United States of America; University of Chicago, United States of America

## Abstract

Ongoing modernization in India has elevated the prevalence of many complex genetic diseases associated with a western lifestyle and diet to near-epidemic proportions. However, although India comprises more than one sixth of the world's human population, it has largely been omitted from genomic surveys that provide the backdrop for association studies of genetic disease. Here, by genotyping India-born individuals sampled in the United States, we carry out an extensive study of Indian genetic variation. We analyze 1,200 genome-wide polymorphisms in 432 individuals from 15 Indian populations. We find that populations from India, and populations from South Asia more generally, constitute one of the major human subgroups with increased similarity of genetic ancestry. However, only a relatively small amount of genetic differentiation exists among the Indian populations. Although caution is warranted due to the fact that United States–sampled Indian populations do not represent a random sample from India, these results suggest that the frequencies of many genetic variants are distinctive in India compared to other parts of the world and that the effects of population heterogeneity on the production of false positives in association studies may be smaller in Indians (and particularly in Indian-Americans) than might be expected for such a geographically and linguistically diverse subset of the human population.

## Synopsis

Genomic studies of human genetic variation are useful for investigating human evolutionary history, as well as for designing strategies for identifying disease-related genes. Despite its large population and its increasing complex genetic disease burden as a result of modernization, India has been excluded from most of the largest genomic surveys.

The authors performed an extensive investigation of Indian genetic diversity and population relationships, sampling 15 groups of India-born immigrants to the United States and genotyping each individual at 1,200 genetic markers genome-wide. Populations from India, and groups from South Asia more generally, form a genetic cluster, so that individuals placed within this cluster are more genetically similar to each other than to individuals outside the cluster. However, the amount of genetic differentiation among Indian populations is relatively small. The authors conclude that genetic variation in India is distinctive with respect to the rest of the world, but that the level of genetic divergence is smaller in Indians than might be expected for such a geographically and linguistically diverse group.

## Introduction

In addition to its use in understanding human evolutionary history, investigation of human genetic variation and population structure is important for the design and analysis of studies that map disease-susceptibility loci. For example, if human genetic disease is largely a consequence of common alleles and haplotypes, identifying common variants in a given population provides a database of predictors that can be tested in that population for association with disease status [1–3]. In examining genetic variants for disease association, knowledge of population structure is important for evading the spurious associations that can be produced by heterogeneity in the ancestry of sampled individuals [4–7].

During the last few decades, the prevalence in India of complex genetic diseases associated with increased life span and with an urban and western lifestyle—including coronary artery disease, non-insulin-dependent diabetes, and metabolic syndrome—has risen considerably and is now greater than in most other populations [8–12]. However, Indian populations have not generally been incorporated into the largest genomic surveys [2,3,13], and thus, a genome-wide catalog of genetic variation important to the design of association studies does not yet exist for India [14]. In addition, the modern studies of the autosomal genome with the most extensive geographical coverage of India have not generally had extensive coverage of non-Indian populations [15–17], making it difficult to place knowledge about Indian genetic diversity in the context of worldwide variation.

**Figure 1:**
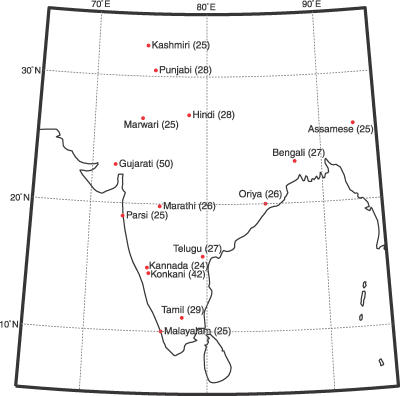
Sample Sizes and Geographic Origins of Samples The latitudes and longitudes used for the various groups are given in Table S2.

To assess both the patterns of genetic variability within India as well as the distinctiveness of Indian variation with respect to that of other groups, we examined autosomal genetic variation at 729 microsatellite and 471 insertion/deletion polymorphisms in a collection of 432 individuals of Indian descent sampled in the United States. These individuals represent 14 groups defined by language, as well as one additional cultural group (Figure 1). Because the study participants were born in India (see Materials and Methods), we refer to the individuals and populations as being “Indian” or “from India”; as we discuss later, it is important to note that because the Indian individuals were sampled in the United States, some biases may be introduced when extrapolating the results to India as a whole. Among the markers, 715 of the microsatellites and 207 of the indels were previously studied in the HGDP-CEPH Human Genome Diversity Cell Line Panel [18–21], enabling comparison of variation in our sample with that in a genetically well-characterized worldwide sample of 53 populations.

## Results/Discussion

**Figure 2:**
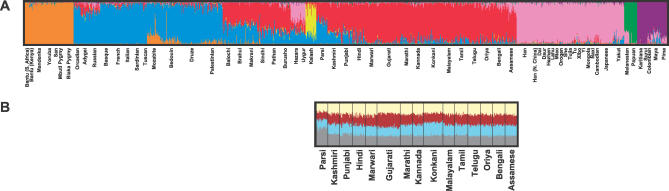
Population Structure Inferred from Microsatellite and Insertion/Deletion Polymorphisms (A) Representative estimate of population structure for 1,384 individuals from worldwide populations, including 432 individuals from India. The plot represents the highest-likelihood run among ten STRUCTURE runs with *K* = 7 clusters. Eight of the other nine runs identified a cluster largely corresponding to India, and five of these eight produced plots nearly identical to the one shown. (B) Representative estimate of population structure for the 432 individuals from India (based on all 1,200 markers). The plot, with *K* = 4 clusters, represents the highest-likelihood run among all 80 runs performed with *K* > 1. None of the 80 runs produced clusters that contained the full ancestry of any particular individual. Across these runs, the clusteredness statistic [21], which measures the extent to which a randomly chosen individual has membership in one as opposed to many clusters, ranged from 0.07 to 0.09.

**Figure 3:**
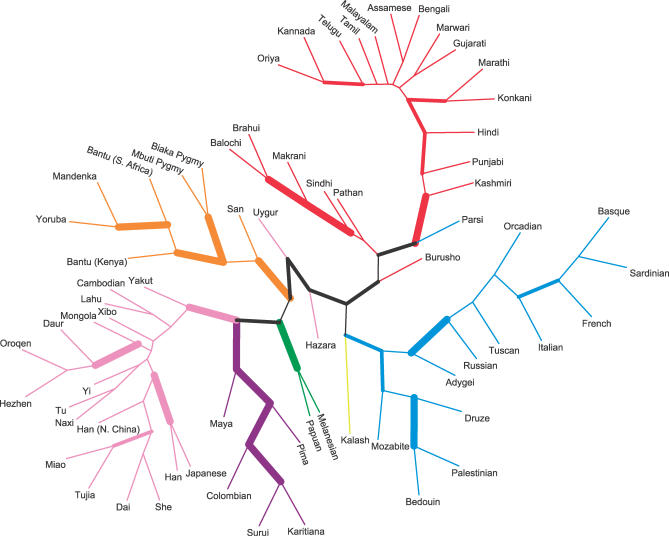
Consensus Neighbor-Joining Tree of Populations The thickest edges have at least 95% bootstrap support, and the edges of intermediate thickness have at least 75% support. If all of the groups subtended by an edge have majority membership in the same cluster in Figure 2A (or only plurality membership in the cases of Hazara , Makrani, and Uygur), the edge is drawn in the same color as was used for the cluster.

Levels of genetic variation in the Indian populations, as measured by microsatellite heterozygosity, are compatible with a general reduction of this genetic variation statistic with increasing distance from sub-Saharan Africa [20,22], ranging from 0.723 to 0.734 across the Indian groups, compared with ranges of 0.747–0.765 in sub-Saharan Africa, 0.722–0.739 in the Middle East and North Africa, 0.718–0.735 in Europe, 0.683–0.737 in the whole of Asia (with the smallest values in East Asia), and 0.515–0.674 in Oceania and the Americas (Table 1). Analysis of population structure in the full sample of individuals via model-based clustering identifies a genetic cluster (a subgroup with increased similarity of genetic ancestry) corresponding largely to the new samples of Indian descent, together with substantial fractions of the inferred ancestry of previously sampled individuals from Pakistan (Figure 2A). This cluster appears consistently when the data are studied using a model whose number of clusters is seven or more and is sometimes present in analyses using fewer clusters. In analyses with seven clusters (the largest number of clusters for which a single clustering solution was observed in a majority of replicates), the remaining six clusters match those previously observed with a set of 377 loci when the Indian data were not available [19]. A distance-based clustering algorithm produces results that are similar to those of the model-based analysis, with 983 of 1,000 bootstrap replicates supporting a grouping of all Indian populations except Parsis (comparatively recent immigrants to India from Persia around 700–800 CE [23]) and with similarly strong support for other major continental groupings (Figure 3).

**Table 1:**
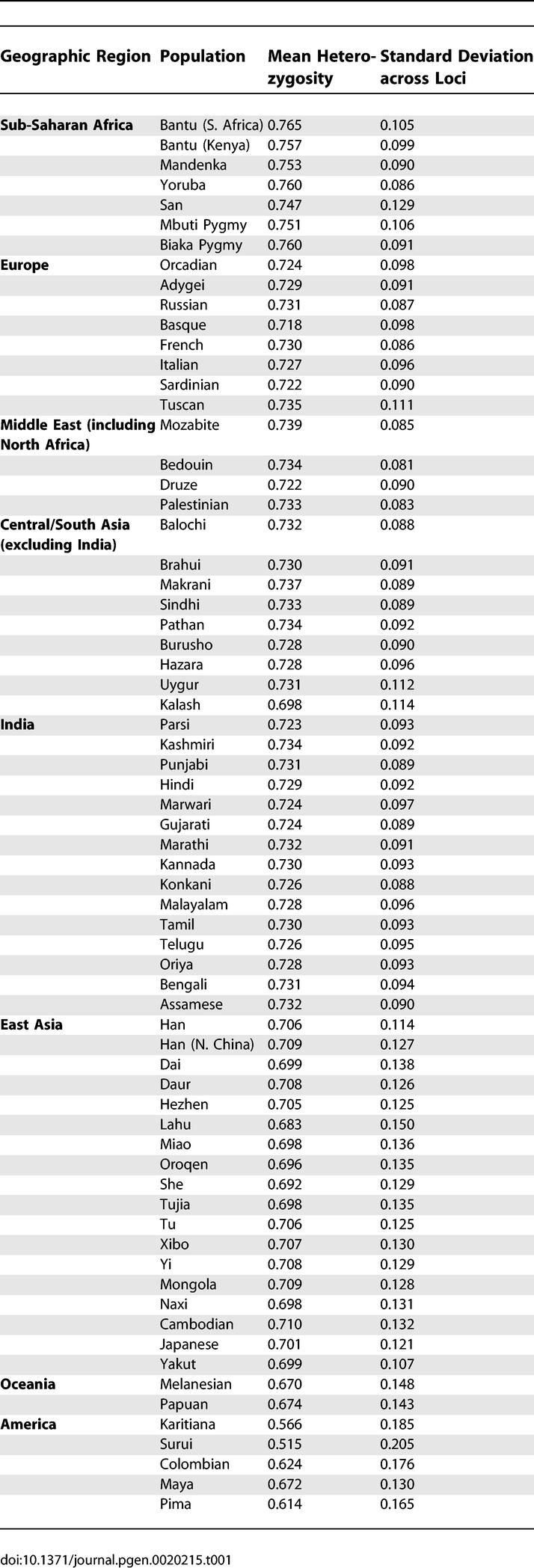
Mean Heterozygosities across 715 Microsatellite Loci

**Figure 4:**
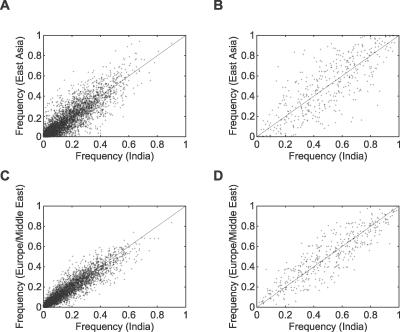
Comparison of Allele Frequencies in India to Allele Frequencies in East Asia and Europe/Middle East (A) East Asia, 8,618 alleles at 715 microsatellites. (B) East Asia, 414 alleles at 207 indels. (C) Europe/Middle East, 8,618 alleles at 715 microsatellites. (D) Europe/Middle East, 414 alleles at 207 indels.

Comparing allele frequencies in the groups from India to those in other geographic regions, allele frequency correlation coefficients are highest for the populations previously studied in Central/South Asia, followed by those of Europe and the Middle East and of East Asia (Table 2). This similarity with Europe and East Asia has been seen in smaller-scale autosomal studies that have incorporated India [24–26]; however, these studies, along with one study of more markers but a smaller number of populations [27], have disagreed somewhat about whether the similarity of India is greater with East Asian populations [24], greater with European populations [26], or about equal between these alternatives [25,27]. We found that allele frequencies in India showed detectably greater similarity to populations in Europe and the Middle East than to those in East Asia (Figure 4). This result is consistent with the fact that the cluster corresponding to India in Figure 2A subdivides a previously obtained cluster corresponding to Europe, the Middle East, and Central/South Asia [19].

**Table 2:**
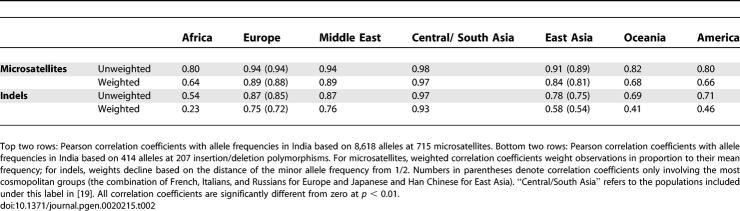
Correlation Coefficients of Allele Frequencies

The noticeable genetic divergence of India from other regions is coupled with low levels of genetic divergence across the subgroups within India. Excluding the relatively divergent Parsi population, *F_st_* in India had similar magnitude to the level of divergence among cosmopolitan groups in Europe and East Asia (Table 3): for the 715 microsatellites genotyped in the worldwide sample, it equaled 0.0038 (see Table 3 for confidence intervals), compared to 0.0046 among French, Italians, and Russians and 0.0048 between Japanese and Han Chinese. For the indels, for which *F_st_* is systematically higher than for microsatellites, the same three comparisons produced 0.0056, 0.0116, and 0.0059, respectively. Considering all populations in India, Europe, and East Asia, microsatellite *F_st_* for India was 0.0049, smaller than the values of 0.0078 for Europe and 0.0110 for East Asia. Similarly, for the indels, India had *F_st_* = 0.0079, whereas Europe and East Asia had *F_st_* = 0.0110 and 0.0190, respectively.

**Table 3:**
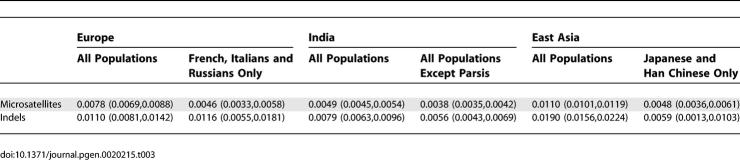
*F_st_* and 95% Confidence Intervals

The low level of genetic divergence in India was reflected by the fact that the STRUCTURE program had difficulty detecting population structure within India (Figure 2B). However, consistent with the fact that *F_st_* was significantly greater than zero across Indian populations (Table 3), 55 of 80 STRUCTURE runs using more than one cluster (*K* > 1) with the Indian genotypes produced higher likelihoods than those that used only one cluster (*K* = 1). This observation, together with the reasonably strong support in the neighbor-joining tree for particular groupings within India, suggests that a detectable amount of population structure does exist in the Indian data and that the addition of more loci might cause clusters corresponding to specific subsets of the Indian sample to become distinguishable. It is noteworthy, however, that in previous analyses of other geographic regions [19,28] using smaller numbers of markers, subclusters have been more easily identifiable elsewhere than was seen here for India with 1,200 markers.

**Figure 5:**
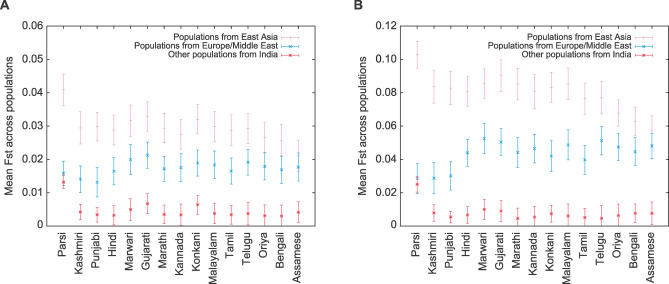
Mean *F_st_* Values for Each Indian Group across Comparisons with 18 Populations from East Asia, 12 Populations from Europe and the Middle East, and the Other 14 Groups from India (A) 715 microsatellites. (B) 207 indels. Error bars denote standard deviations across comparisons.

**Figure 6:**
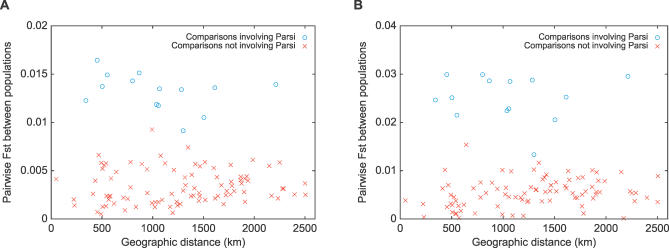
Relationship of *F_st_* and Geographic Distance for Pairs of Groups from India (A) 715 microsatellites (correlation coefficient of −0.10 [*p* = 0.32], or 0.09 [*p* = 0.41] if comparisons involving the Parsi group are excluded). (B) 207 indels (correlation coefficient of −0.02 [*p* = 0.84], or 0.28 [*p* = 0.007] when excluding comparisons involving the Parsi group). A complete list of pairwise values of *F_st_* is contained in Table S3.

Figure 5 illustrates both the relatively low levels of genetic differentiation among populations within India and the comparatively higher levels of divergence between Indian populations and those of Europe and the Middle East, as well as with those of East Asia. Consistent with geography and with the analysis in Figure 2A, among the Indian populations, populations from the northwest part of India, including the Kashmiri and Punjabi groups, had the greatest similarity to the populations from Europe and the Middle East. Populations from eastern India, including the Assamese and Bengalis, had the greatest similarity to the populations from East Asia. The only population whose *F_st_* values within India substantially overlapped those of either Europe/Middle East or East Asia was the Parsi population. *F_st_* values for the Parsis were similar within India and with populations from Europe and the Middle East, in agreement with their likely origins and their similar membership in the blue and red clusters in Figure 2A (52.4% for the blue cluster, 45.3% for the red cluster). In general, *F_st_* between pairs of populations within India did not show a strong correlation with geographic distance (Figure 6). The correlation was greater when excluding pairs involving the Parsi population, but remained considerably smaller than has been seen for other sets of worldwide populations [20,21].

Compared to groups that speak Indo-European languages, the groups in our study that speak Dravidian languages (Kannada, Malayalam, Tamil, and Telugu) did not show noticeably different patterns of pairwise *F_st_* values, and in particular, they did not show a greater *F_st_* from populations of Europe and the Middle East (Figure 5). Although a process of ancient admixture with indigenous Dravidian speakers by Indo-European populations originating to the west of India might have been expected to result in an elevated genetic distance from modern Dravidians to European and Middle Eastern populations, our analysis does not find evidence of such an admixture process. However, the admixture scenario is not directly contradicted: the data are compatible with a view in which the admixture occurred in such a manner that at its conclusion, similar contributions of ancestral Dravidians were present in the precursors of the modern Dravidian-speaking and non-Dravidian-speaking groups of our study.

**Figure 7:**
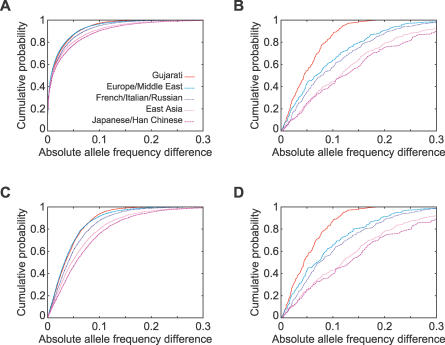
Cumulative Distribution Function for the Absolute Allele Frequency Difference between Various Populations and (Non-Gujarati) Indians (A) All alleles at 715 microsatellites. (B) All alleles at 207 indels. (C) Common alleles at 715 microsatellites (alleles whose frequencies average more than 0.05 in the two groups). (D) Common alleles at 207 indels (alleles whose frequencies have a mean above 0.05 and below 0.95 in the two groups).

**Figure 8:**
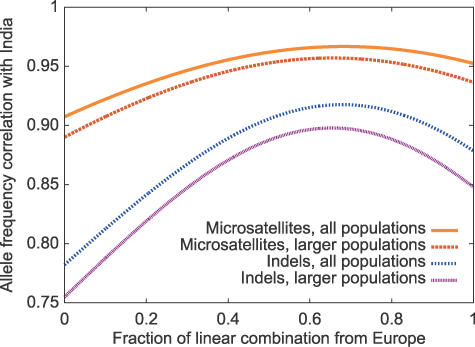
Correlation Coefficient of Allele Frequencies in India with Linear Combinations of the Allele Frequencies in Europe and East Asia, as a Function of the Fraction of the Linear Combination Drawn from Europe Graphs with “all populations” compare the frequencies in the pooled Indian sample with linear combinations of the pooled European/Middle Eastern sample and the pooled East Asian sample; graphs with “larger populations” exclude the Parsi group from the Indian sample and utilize only the pooled French, Italian, and Russian genotypes in Europe and the pooled Han Chinese and Japanese genotypes in East Asia. The maxima of the four graphs, from top to bottom, occur at (0.685, 0.967), (0.656, 0.957), (0.679, 0.918), and (0.654, 0.898), respectively. The analysis considers all alleles found worldwide at the 715 microsatellites and 207 indels.

The relatively high correlation coefficients of allele frequencies between European or Middle Eastern populations and Indian populations (0.94 and 0.87 for microsatellites and indels, respectively) suggest that European allele frequencies are often reasonably predictive of frequencies in India, particularly for microsatellites (Figure 7A and 7C). The correlations are increased by using a linear combination of allele frequencies with ∼2/3 contribution from Europe/Middle East and ∼1/3 contribution from East Asia (Figure 8). At the same time, however, the separate cluster for India in population structure analysis indicates that allele frequencies in India are distinctive, so that predictions obtained based on European and East Asian groups cannot fully explain allele frequencies in Indian populations. This comment applies particularly for the indels (Figure 7B and 7D); for example, 40% of indel alleles have an absolute difference in frequency >0.1 between Europe and India. Additionally, it is noteworthy that because common alleles have greater potential for frequency differences than do rare alleles, the frequency divergence may be larger for more frequent alleles; when a correlation coefficient placing a larger weight on common alleles is used, the allele frequency correlations between Europe and India decline to 0.89 and 0.75 for microsatellites and indels, respectively (Table 2).

Although India (together with several populations from Pakistan) was observed to be distinctive with respect to the remainder of the sample, at the same time, genetic differentiation within India was found to be relatively small. Thus, inclusion of a single population from India in a genome-wide survey of variation is likely to increase the accuracy of predictions made about frequencies of genetic variants in India, in comparison with those that could be made based on frequencies in groups that are currently well studied. For example, for 87% of the indels, the Gujarati sample had an absolute allele frequency difference of less than 0.1 from the non-Gujarati Indians, whereas the corresponding values were 57% for a mixture of French, Italians, and Russians and 43% for a mixture of Japanese and Han Chinese (Figure 7B).

Because of the relationship between genetic heterogeneity and the production by population structure of spurious associations in case-control association studies [4,5,29], an additional consequence of the low level of Indian genetic differentiation is that for phenotypes whose patterns of variation across subgroups are similar in different parts of the world, false positives due to population structure in populations with mixed ancestry across India are likely to occur with a similar or smaller frequency as in population mixtures from East Asia or Europe (e.g., European-Americans). However, this claim should not be taken to imply that spurious associations in Indian samples will be absent or unimportant, as the effects of even extremely low levels of population structure can substantially elevate the false-positive rate when samples increase in size [4–6].

Note that with the exception of the Parsis, this investigation incorporated individuals into the study based on primary spoken language, considering 14 of the most frequently spoken languages of India (all of which, except Marwari, are among the 22 current official indigenous languages of India). Although different studies have sometimes reached divergent conclusions about the magnitude of Indian differentiation and its determinants [15–17,30–38], it is possible that in India, differentiation across groups is larger than was seen here, but is based not on language, but on other variables, such as geography or caste [15,16,27,39]. However, as the observed correlation of genetic and geographic distance is small, our study does not suggest an easily interpreted geographic effect. The lack of a straightforward geographic effect was reflected in an analysis of the Indian data with the program TESS [40], a clustering program similar to STRUCTURE that has the additional feature of incorporating prior information about the spatial structure of the sample in the identification of clusters. When geography is an important determinant of population structure, the use of the spatial information assists in identifying the clusters, and TESS is expected to produce a stronger degree of clustering than that obtained with STRUCTURE [40]. In replicate analyses with TESS, however, similar to the STRUCTURE analyses, distinctive clusters were not found, except for the somewhat inconsistent identification of a Parsi cluster (unpublished data). Although caste may well be an important factor underlying genetic structure, when considering a subset consisting of 77 individuals from three language groups for which caste information was available, *F_st_* between castes (0.0017 for the 715 microsatellites, with 95% confidence interval [0.0008, 0.0026]; 0.0026 for the 207 indels, with 95% confidence interval [−0.0008, 0.0064]) remained small, having a similar magnitude to *F_st_* among the three language groups (0.0015 for microsatellites, with 95% confidence interval [0.0007, 0.0025]; 0.0018 for indels, with 95% confidence interval [−0.0013, 0.0050]). However, these slightly larger *F_st_* values between castes compared with among language groups (despite considerable overlap in confidence intervals) suggest that a more complete investigation of the relative importance of caste and language would be worthwhile.

It is also possible that genetic divergence would have been larger if Indian tribal populations had been included along with the relatively cosmopolitan groups we examined or if Indian representatives of other language families besides Dravidian and Indo-European had been included [15,30,32,37,38,41,42]. For example, a recent study of 15 microsatellite loci in 54 groups [17], including tribal populations, identified population subclusters within India and obtained *F_st_* = 0.018, considerably greater than the value seen here. By necessity, however, our study required sampling in the United States in order to allow high-throughput genotyping of the large number of markers that we investigated, and it was not possible to include groups without an appreciable presence in the United States. Although our sample is likely to be reasonably representative of first-generation individuals of Indian descent currently located in the United States, such individuals likely do not provide a random sample of the source populations in India, as urban and relatively mobile populations and populations of higher caste and socioeconomic status are overrepresented among immigrants. Thus, if variables such as caste and socioeconomic status do play important roles in producing genetic structure, more genetic differentiation would certainly be expected for a sample of the same linguistic groups in India compared to what we have seen in the United States. Additionally, if higher caste is correlated with a European or western Asian component of ancestry, a sample in the United States may be biased towards finding a greater similarity of populations from India to those of the Europe/Middle East rather than to those of East Asia. However, despite the limitations of our study, it remains significant that in the largest genomic analysis of India performed to date, across a broad range of language and geography within India, such a low level of genetic divergence was observed.

## Materials and Methods

### Sampled individuals.

Individuals of Indian descent were enrolled into the study by PIP in various cities in the United States, primarily Houston, Texas, and New Brunswick, New Jersey. Nearly all of these individuals were first-generation immigrants, and particular care was taken to ensure that all four grandparents of each individual spoke the same language and originated from the same state in India. For many of them, other demographic information was gathered, including caste and endogamic group affiliations if applicable, religious sect, and brief medical history. All individuals gave their informed consent for participation, and the study was approved by the Institutional Review Board of Baylor College of Medicine. Among the 673 DNA individuals sampled, three sets of duplicate individuals were later identified (658–24 [kindred 658 individual 24] and 458–147; 425–1 and 425–491; 298–43 and 470–43), and one member of each pair was discarded (458–147, 425–491, and 470–43). The sample was designed so that when subdividing participants by their primary spoken Indian language, 14 languages, each having a relatively localized distribution within India, would be well represented. One additional cultural group, the Parsis, was also sampled. As the number of Gujaratis sampled (279 individuals) was large in comparison with sample sizes for other language groups, this study utilized a subset consisting of 432 individuals in the 15 groups (Figure 1). Together with computations of the proportions of alleles shared identical in state between pairs of individuals, the RELPAIR program [43,44] was used to verify that none of the 432 individuals studied were related at a level closer than first cousins. Among the 432 individuals, 428 were first-generation immigrants born in the Indian subcontinent who have resided in the United States for less than ∼40 years. The remaining four individuals (407–123, 442–90, 445–135, and 510–79), all from the Gujarati group, were second-generation immigrants whose parents followed strict endogamic practices.

For the Kannada, Tamil, and Telugu groups, caste information was available on nearly all individuals. Because most castes were represented by relatively few individuals, analysis of caste in these samples utilized a subdivision into Brahmins (15 Kannada, 21 Tamil, eight Telugu) and non-Brahmins (nine Kannada, six Tamil, 18 Telugu).

### DNA preparation and cell lines.

Blood (20 ml) was collected from each individual. DNA was isolated using the Puregene DNA isolation kit (Gentra Systems, http://www.gentra.com) from 10 ml of blood. The remainder was used to establish a lymphoblastoid cell line by standard procedures [45].

### Markers.

Each individual was genotyped for 1,200 polymorphisms spread across all 22 autosomes: 471 insertion/deletion polymorphisms and 729 microsatellites. The microsatellites were drawn from Marshfield Screening Sets 13 and 52 [46], and the insertion/deletion markers were drawn from Marshfield Screening Set 100 [47]. The proportion of missing data in the individuals genotyped from India was 2.3% for the microsatellites and 1.8% for the indels.

### Combined dataset including the HGDP-CEPH Human Genome Diversity Cell Line Panel.

Of the markers typed in the Indian sample, 932 of them (207 indels and 725 microsatellites) had been previously typed in the HGDP-CEPH Human Genome Diversity Cell Line Panel [18–21]. However, for some microsatellites, either a change in primer length or position occurred between the time that the HGDP-CEPH samples were genotyped (2002) and the time that the Indian individuals were genotyped (2004), or a systematic change occurred in the algorithm by which allele size was determined from raw genotyping products, or both. In cases in which the primer changed, allele sizes from one of the two datasets were adjusted by the appropriate length in order to align the two lists of allele sizes (Table S1). Two loci for which the allele size shift was not possible to determine, ATA29C07 and GGAA10C09, were excluded from consideration.

To identify systematic changes in allele calling (the procedure by which allele size was obtained from genotyping products), for each locus, the allele sizes of one dataset were translated by a constant, and the *G* test statistic of independence [48] between allele frequencies and dataset (older HGDP-CEPH dataset versus newer Indian dataset) was then computed. Considering all possible constants for translation of allele sizes, the one that minimized the *G* statistic was determined. In implementing the *G* test, all 673 typed Indians were compared to only the Pakistanis in the HGDP-CEPH panel, a collection of 198 individuals (all individuals in the panel from Pakistan except the sample duplicates 111 and 220). Of the 932 loci considered, 923 had *G* < 45, and the other nine had *G* > 100. These nine loci (AAT228, ATA27C11, GATA164B08, GATA21D04, GATA86E02, GGAA4B09, GGAA10C09, GTT035, and UT5029) were excluded from consideration. As GGAA10C09 had already been excluded on the grounds of an inability to determine the shift in allele sizes, 922 loci remained for the combined analysis with the HGDP-CEPH panel, 207 indels and 715 microsatellites. Among the 715 shared microsatellites, primers had changed between datasets for 133, while for the remaining 582 there was no change. All analysis utilized these 922 loci except where otherwise specified.

The set of 952 individuals used here from the HGDP-CEPH Human Genome Diversity Cell Line Panel [18] is the “H952” subset of the original panel, and it omits relative pairs, sample duplicates, and two individuals who were extremely atypical for their respective populations [49].

### Geographic computations.

Geographic coordinates were based on the state of origin within India for sampled individuals (Table S2). Seven of the individuals reported ancestry outside India, including three from Pakistan (235–328 in the Hindi group and 86–334 and 196–337 in the Punjabi group) and four Bengalis from Bangladesh (14–270, 206–267, 217–261, and 225–275). For several of the Indian groups, multiple states were reported across the set of individuals sampled. In these cases the coordinates used for the group were the inverse sine of the mean of the sines of the latitudes for included individuals and the mean of the longitudes. Geographic distances between pairs of groups were computed as in [21]. Excluding the Hindi and Konkani groups, the total number of individuals who were not from the state with highest representation in their population was 18, distributed across several groups; for the Hindi and Konkani groups, the numbers of individuals not from the modal state were 18 and 16, respectively. Thus, to avoid this geographic heterogeneity, correlations of *F_st_* and geographic distance were computed with and without the Hindi and Konkani groups. For microsatellites, the correlation coefficient between *F_st_* and geographic distance was −0.11 (*p* = 0.32) with Hindi and Konkani excluded, and 0.13 (*p* = 0.28) with Hindi, Konkani, and Parsi excluded; for indels, these two correlations equaled −0.04 (*p* = 0.74) and 0.31 (*p* = 0.01), respectively. These values do not differ substantially from those obtained when the Hindi and Konkani groups were retained in the analysis (Figure 6).

### Population-genetic analysis.

Expected heterozygosity was computed using the sample size–corrected estimator [50]. *F_st_* was estimated as in [51] (equation 5.3), with 95% confidence intervals based on 1,000 bootstraps across loci. Weighted allele frequency correlations for pairwise population comparisons were obtained by assigning each microsatellite allele a weight of (*p*+*q*)/2, where *p* and *q* denote the frequency of the allele in the two populations. Thus, alleles were weighted linearly in proportion to their mean frequency. For indels, we used a weight of 1−2|(*p*+*q*)/2−1/2|, so that indel alleles were weighted linearly based on nearness of the minor (or major) allele frequency to 1/2. Due to its symmetry around 1/2, this scheme gives equal weight to the minor and major alleles of biallelic markers. The *p*-value for the null hypothesis that a weighted correlation coefficient equaled zero was obtained as the fraction of bootstrap replicates (with resampling performed across alleles) in which the correlation coefficient was negative.

Cluster analysis of genotypes utilized the STRUCTURE software package [52,53]. STRUCTURE runs were based on a burn-in period of 20,000, followed by 10,000 iterations from which estimates were obtained. For each value of *K* from 1 to 9, ten replicates were performed with the Indian individuals only and also with the combined Indian and diversity panel individuals. All runs used the *F* model for correlations of allele frequencies across clusters [53]. Additional cluster analysis was performed with TESS [40,54], a program that incorporates spatial information when identifying clusters of individuals. In this analysis we considered several values of the spatial dependence parameter ranging from 0 to 1. Runs were performed with a variety of different lengths, ranging from shorter runs of 5,000 total iterations to runs comparable in length to those used in the analysis with STRUCTURE.

The neighbor-joining tree [55], obtained using greedy consensus as implemented in PHYLIP [56], was based on the proportion-of-shared-alleles distance matrix [57], with 1,000 bootstraps across loci. Alternative genetic distances produced similar results (unpublished data). Both the STRUCTURE and neighbor-joining analyses of the 1,384 Indian and non-Indian individuals used all 922 markers that overlapped between the Indian and non-Indian samples.

## Supporting Information

Table S1 Amount by Which Allele Sizes Were Translated to Make the HGDP-CEPH and Indian Microsatellite Data Comparable (21 KB PDF)

Table S2 Latitudes and Longitudes Used for the Samples from India (8 KB PDF)

Table S3 Pairwise Values of *F_st_* for Indian Populations (12 KB PDF)

## Abbreviations

CEPH: Centre d'Etude du Polymorphisme Humain
HGDP: Human Genome Diversity Project
