# Supplementary material for: Low Levels of Genetic Divergence across Geographically and Linguistically Diverse Populations from India
Source: PLoS Genet. 2006 Dec 22;2(12):e215. doi: 10.1371/journal.pgen.0020215 (PMC1713257; doi:10.1371/journal.pgen.0020215)
Supplement: Supplementary file 1 [file pgen.0020215.st001.pdf]

Table S1. Amount by which allele sizes were translated to make the HGDP-CEPH and Indian microsatellite data comparable.

	Name of locus in data file for India	Name of locus in data file for HGDP-CEPH	Amount to subtract from allele sizes in India data for combined analysis with HGDP-CEPH data
1	ATA11D10M_06_Q	ATA11D10	1
2	ATA18A07M_09	ATA18A07	1
3	ATA20G07M_05_P	ATA20G07	1
4	ATA22G07P_06_Q	ATA22G07	7
5	ATA23A10M_05_Q	ATA23A10	1
6	ATA25E07M_01_P	ATA25E07	1
7	ATA25F09M_12	ATA25F09	1
8	ATA27A06P_12	ATA27A06	7
9	ATA27C07P_04	ATA27C07	7
10	ATA27D04P_02_P	ATA27D04	7
11	ATA28G05P_15	ATA28G05	7
12	ATA29A06P_12	ATA29A06	7
13	ATA29C07L_01_Q	ATA29C07	?
14	ATA29G03Z_14	ATA29G03	0
15	ATA31G11P_10	ATA31G11	7
16	ATA33B03Z_11	ATA33B03	0
17	ATA34E08N_11	ATA34E08	0
18	ATA42G12M_01_P	ATA42G12	1
19	ATA55A11N_16	ATA55A11	0
20	ATA58A02P_17	ATA58A02	7
21	ATA59H06Z_09	ATA59H06	0
22	ATA5A04N_10	ATA5A04	0
23	ATA5A09N_13	ATA5A09	0
24	ATA78D02N_17	ATA78D02	0
25	ATA82B02N_18	ATA82B02	0
26	ATA9B04N_11	ATA9B04	0
27	ATC6A06M_17	ATC6A06	1
28	GAAT1A4N_08	GAAT1A4	0
29	GAAT2C03P_17	GAAT2C03	7
30	GATA115E01N_10	GATA115E01	0
31	GATA116B01N_02_P	GATA116B01	0
32	GATA117D01N_11	GATA117D01	0
33	GATA11A11P_05_Q	GATA11A11	7
34	GATA11C06N_16	GATA11C06	0
35	GATA11C08P_13	GATA11C08	7
36	GATA11E02N_06_P	GATA11E02	0
37	GATA121A08N_10	GATA121A08	0
38	GATA124C08N_01_P	GATA124C08	0
39	GATA128C02M_03_P	GATA128C02	1
40	GATA129B03N_20	GATA129B03	0
41	GATA129D11N_21	GATA129D11	0
42	GATA12A07N_01_P	GATA12A07	0
43	GATA133A08Q_01_P	GATA133A08	4
44	GATA137H02N_07_P	GATA137H02	0
45	GATA145D10N_05_P	GATA145D10	0
46	GATA151F03N_15	GATA151F03	0

47	GATA163B10N_06_P	GATA163B10	0
48	GATA164B08P_03_P	GATA164B08	7
49	GATA165G02M_06_Q	GATA165G02	1
50	GATA177C03N_18	GATA177C03	0
51	GATA178F11Z_18	GATA178F11	-67
52	GATA178G09M_02_Q	GATA178G09	1
53	GATA185C06Z_18	GATA185C06	17
54	GATA185H04N_17	GATA185H04	0
55	GATA187D09N_09	GATA187D09	0
56	GATA189C06M_07_Q	GATA189C06	1
57	GATA197B10P_15	GATA197B10	7
58	GATA198B05N_22	GATA198B05	0
59	GATA22F09P_16	GATA22F09	7
60	GATA22F11NZ_03_Q	GATA22F11	-58
61	GATA22G05M_04	GATA22G05	1
62	GATA23B01N_19	GATA23B01	0
63	GATA23C03P_13	GATA23C03	7
64	GATA23D03N_02_Q	GATA23D03	0
65	GATA23E06L_11	GATA23E06	3
66	GATA23F06L_11	GATA23F06	3
67	GATA24D12P_07_P	GATA24D12	7
68	GATA24H01N_04	GATA24H01	0
69	GATA26D02M_12	GATA26D02	1
70	GATA26E03M_08	GATA26E03	1
71	GATA26G09P_01_P	GATA26G09	7
72	GATA27C08P_03_P	GATA27C08	7
73	GATA28D01M_11	GATA28D01	1
74	GATA29A05P_01_P	GATA29A05	7
75	GATA29B01L_19	GATA29B01	-1
76	GATA29F06Z_20	GATA29F06	1
77	GATA30A03N_14	GATA30A03	0
78	GATA30D09N_07_Q	GATA30D09	0
79	GATA30E06P_02_Q	GATA30E06	7
80	GATA3C02M_03_Q	GATA3C02	1
81	GATA3H06M_05_Q	GATA3H06	1
82	GATA41G07M_07_P	GATA41G07	1
83	GATA42H02P_04	GATA42H02	8
84	GATA43H01M_14	GATA43H01	1
85	GATA43H03N_13	GATA43H03	0
86	GATA44F10P_19	GATA44F10	7
87	GATA45B10N_20	GATA45B10	0
88	GATA49C09N_17	GATA49C09	0
89	GATA49D12N_12	GATA49D12	0
90	GATA50C03N_15	GATA50C03	0
91	GATA51B02M_13	GATA51B02	1
92	GATA52A04M_02_Q	GATA52A04	1
93	GATA5B02M_04	GATA5B02	1
94	GATA62F03M_09	GATA62F03	1
95	GATA63A03N_15	GATA63A03	0
96	GATA63D12P_12	GATA63D12	7
97	GATA65C03M_02_Q	GATA65C03	1
98	GATA69E12M_02_P	GATA69E12	1
99	GATA6B02P_08	GATA6B02	7

100	GATA6B09P_11	GATA6B09	7
101	GATA72G09N_04	GATA72G09	0
102	GATA72G11N_02_P	GATA72G11	0
103	GATA73D10L_07_P	GATA73D10	-4
104	GATA73F01M_15	GATA73F01	1
105	GATA73H09N_12	GATA73H09	0
106	GATA74E02Z_14	GATA74E02	0
107	GATA7C06M_05_P	GATA7C06	1
108	GATA7D01ZP_04	GATA7D01	-37
109	GATA81C04M_09	GATA81C04	1
110	GATA81D12M_16	GATA81D12	1
111	GATA85A04M_12	GATA85A04	1
112	GATA86E02P_02_P	GATA86E02	7
113	GATA87E02N_09	GATA87E02	0
114	GATA88H02N_15	GATA88H02	0
115	GATA89G08Z_05_Q	GATA89G08	-31
116	GATA8B05M_03_P	GATA8B05	1
117	GATA8G10M_08	GATA8G10	1
118	GATA90D07N_11	GATA90D07	0
119	GATA91H06M_12	GATA91H06	1
120	GGAA10C09P_14	GGAA10C09	7
121	GGAA10F06M_22	GGAA10F06	1
122	GGAA17G05P_11	GGAA17G05	7
123	GGAA20C10M_08	GGAA20C10	1
124	GGAA20G10M_02_P	GGAA20G10	1
125	GGAA21G11L_14	GGAA21G11	2
126	GGAA22G10N_01_Q	GGAA22G10	0
127	GGAA23C05N_10	GGAA23C05	0
128	GGAA29H03N_13	GGAA29H03	0
129	GGAA2F11N_10	GGAA2F11	0
130	GGAA3A07M_01_P	GGAA3A07	1
131	GGAA4B09N_03_P	GGAA4B09	?
132	GGAA6D03N_07_Q	GGAA6D03	0
133	GGAT3H10M_06_P	GGT3H10	1
134	SRAP_02_P	SRA	7
135	UT1355Z_21	UT1355	3
136	UT2095M_11	UT2095	1
137	UT7129L_08	UT7129	-1
138	UT721M_08	UT721	1

Only those markers for which there was a change in primers are shown; this includes cases in which the primers changed, but the change did not affect the allele sizes. Markers highlighted in yellow were excluded from combined analysis of the HGDP-CEPH and India data, either because the appropriate allele size shift could not be determined, or because a systematic change in allele calling occurred.
