# Supplementary material for: Low Levels of Genetic Divergence across Geographically and Linguistically Diverse Populations from India
Source: PLoS Genet. 2006 Dec 22;2(12):e215. doi: 10.1371/journal.pgen.0020215 (PMC1713257; doi:10.1371/journal.pgen.0020215)
Supplement: Supplementary file 2 [file pgen.0020215.st002.pdf]

Table S2. Latitudes and longitudes used for the samples from India.

Language group	Sample size	Latitude (degrees north)	Longitude (degrees east)
Assamese	25	26	93
Bengali	27	23.25766454	87.66666667
Gujarati	50	23	72
Hindi	28	26.98091726	78.39357143
Kannada	24	15	75
Kashmiri	25	32.44	74.54
Konkani	42	14.57081522	75.13095238
Malayalam	25	10	76.25
Marathi	26	19.81684261	75.94230769
Marwari	25	26.67171131	74.264
Oriya	26	20	85
Parsi	25	19	72.8
Punjabi	28	30.47159766	75.29071429
Tamil	29	11.10167224	77.94827586
Telugu	27	15.92575810	79.62962963
