# Supplementary material for: Low Levels of Genetic Divergence across Geographically and Linguistically Diverse Populations from India
Source: PLoS Genet. 2006 Dec 22;2(12):e215. doi: 10.1371/journal.pgen.0020215 (PMC1713257; doi:10.1371/journal.pgen.0020215)
Supplement: Supplementary file 3 [file pgen.0020215.st003.pdf]

Table S3. Pairwise values of F_{st} for Indian populations.

	Assam.	Beng.	Gujar.	Hindi	Kann.	Kashm.	Konk.	Malaya.	Mara.	Marw.	Oriya	Parsi	Punj.	Tamil	Telu.
Assamese		0.30	**0.97**	**0.58**	**0.57**	**0.79**	**0.53**	**0.89**	0.43	**1.01**	**0.46**	**2.95**	**0.57**	**0.54**	0.11
Bengali	**0.16**		**0.69**	**0.51**	**0.64**	**0.81**	**0.99**	**0.97**	**0.66**	**0.86**	0.25	**2.53**	**0.70**	0.44	**0.44**
Gujarati	**0.55**	**0.53**		**0.76**	**0.62**	**0.99**	**1.01**	**0.76**	0.38	**0.70**	**0.82**	**2.99**	**0.75**	**0.70**	**0.45**
Hindi	**0.27**	**0.12**	**0.59**		0.37	**0.64**	**0.92**	**0.51**	**0.54**	**0.63**	**0.65**	**2.28**	0.32	0.26	0.35
Kannada	**0.21**	**0.21**	**0.52**	**0.24**		**0.56**	**0.38**	0.02	0.11	**1.02**	0.06	**2.51**	0.44	0.11	0.14
Kashmiri	**0.44**	**0.24**	**0.66**	**0.22**	**0.40**		**0.78**	0.35	**0.56**	**1.54**	**0.85**	**2.06**	0.04	0.44	**0.56**
Konkani	**0.58**	**0.43**	**0.93**	**0.57**	**0.41**	**0.61**		**0.46**	0.17	**1.16**	**0.53**	**2.15**	**0.51**	**0.50**	0.29
Malayalam	**0.25**	**0.18**	**0.59**	**0.28**	**0.23**	**0.37**	**0.56**		0.07	**0.67**	0.38	**2.85**	0.37	0.31	-0.12
Marathi	**0.37**	**0.16**	**0.58**	**0.12**	**0.24**	**0.32**	**0.42**	**0.20**		0.45	0.26	**2.46**	0.40	0.09	-0.22
Marwari	**0.44**	**0.24**	**0.66**	**0.26**	**0.29**	**0.49**	**0.74**	**0.40**	**0.33**		**0.61**	**2.86**	**1.02**	**0.86**	**0.60**
Oriya	**0.18**	0.07	**0.46**	**0.17**	0.06	**0.29**	**0.51**	**0.19**	**0.27**	**0.35**		**2.88**	**0.57**	0.37	0.12
Parsi	**1.39**	**1.36**	**1.64**	**1.18**	**1.37**	**1.05**	**1.49**	**1.35**	**1.23**	**1.51**	**1.34**		**1.33**	**2.24**	**2.99**
Punjabi	**0.29**	**0.24**	**0.57**	0.05	**0.25**	**0.14**	**0.56**	**0.31**	**0.26**	**0.40**	**0.20**	**0.91**		0.01	**0.53**
Tamil	**0.32**	**0.15**	**0.55**	**0.25**	**0.13**	**0.25**	**0.58**	**0.20**	**0.16**	**0.39**	**0.14**	**1.19**	**0.24**		0.27
Telugu	**0.34**	**0.12**	**0.58**	**0.18**	**0.13**	**0.42**	**0.52**	**0.21**	**0.24**	**0.43**	0.09	**1.43**	**0.35**	**0.20**	

The F_{st} estimates equal the values shown divided by a factor of 100. Values for microsatellites and indels are shown below and above the diagonal, respectively. Estimates for which the bootstrap 95% confidence interval did not overlap zero are shown in bold.
